# Incentives for retaining and motivating health workers in Pacific and Asian countries

**DOI:** 10.1186/1478-4491-6-18

**Published:** 2008-09-15

**Authors:** Lyn N Henderson, Jim Tulloch

**Affiliations:** 1 Australian Agency for International Development (AusAID) Canberra, Australia

## Abstract

This paper was initiated by the Australian Agency for International Development (AusAID) after identifying the need for an in-depth synthesis and analysis of available literature and information on incentives for retaining health workers in the Asia-Pacific region. The objectives of this paper are to:

1. Highlight the situation of health workers in Pacific and Asian countries to gain a better understanding of the contributing factors to health worker motivation, dissatisfaction and migration.

2. Examine the regional and global evidence on initiatives to retain a competent and motivated health workforce, especially in rural and remote areas.

3. Suggest ways to address the shortages of health workers in Pacific and Asian countries by using incentives.

The review draws on literature and information gathered through a targeted search of websites and databases. Additional reports were gathered through AusAID country offices, UN agencies, and non-government organizations.

The severe shortage of health workers in Pacific and Asian countries is a critical issue that must be addressed through policy, planning and implementation of innovative strategies – such as incentives – for retaining and motivating health workers. While economic factors play a significant role in the decisions of workers to remain in the health sector, evidence demonstrates that they are not the only factors. Research findings from the Asia-Pacific region indicate that salaries and benefits, together with working conditions, supervision and management, and education and training opportunities are important. The literature highlights the importance of packaging financial and non-financial incentives.

Each country facing shortages of health workers needs to identify the underlying reasons for the shortages, determine what motivates health workers to remain in the health sector, and evaluate the incentives required for maintaining a competent and motivated health workforce. Decision-making factors and responses to financial and non-financial incentives have not been adequately monitored and evaluated in the Asia-Pacific region. Efforts must be made to build the evidence base so that countries can develop appropriate workforce strategies and incentive packages.

## Review

### Health worker shortages in Pacific and Asian countries

The severe shortage of health workers in Pacific and Asian countries is a critical issue that must be addressed as an integral part of strengthening health systems. Health workers are vital to health systems but are often neglected. Factors that contribute to the shortage of skilled health workers include a lack of effective planning, limited health budgets, migration of health workers, inadequate numbers of students entering and/or completing professional training, limited employment opportunities, low salaries, poor working conditions, weak support and supervision, and limited opportunities for professional development. The shortage of workers often results in inappropriate skill mixes in the health sector as well as gaps in the distribution of health workers. This is especially so in rural and remote areas where the provision of services is difficult because of limited health budgets and scattered populations living in isolated villages or islands.

**Figure 1:**
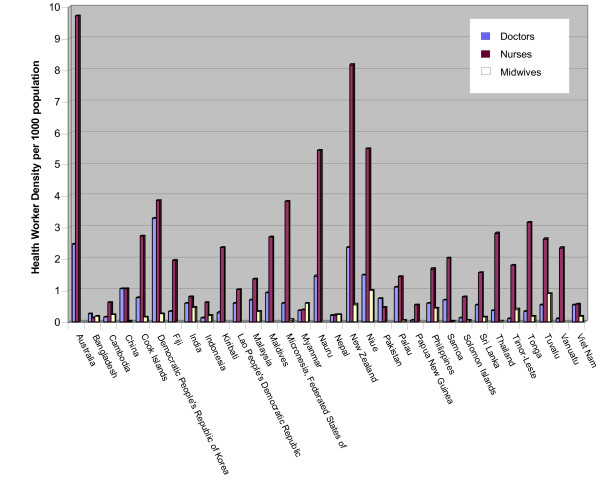
**Density of health workers**. Source: WHO Global Atlas of the Health Workforce (created on 4 July 2007) .

The magnitude of the shortage can be seen in health worker density rates and workforce vacancy rates. Its impact is reflected in health system performance indicators, including maternal and child health indicators, which correlate with health worker density [1]. A threshold of 2.5 health workers (including doctors, nurses and midwives) per 1000 people has been recommended by the Joint Learning Initiative on Human Resources for Health in order to achieve a package of essential health interventions and the health-related Millennium Development Goals [2]. Several countries in Asia and the Pacific fall well below this threshold (Figure 1). For example, Vietnam averages just over one health provider per 1000 people, but this figure hides considerable variation. In fact, 37 of Vietnam's 61 provinces fall below this national average, while one province counts almost four health service providers per 1000 [3].

**Figure 2:**
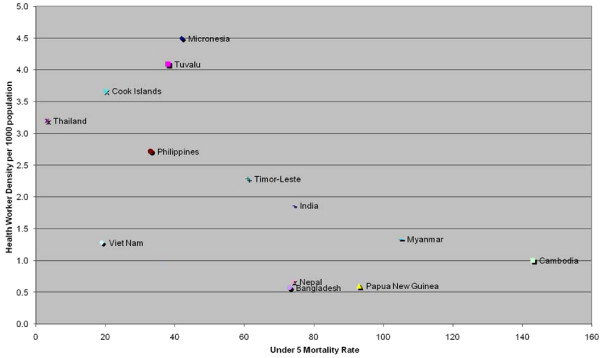
**Density of health workers and child mortality**. Source: WHO Global Atlas of the Health Workforce , and UNICEF Monitoring & Statistics  (accessed and created 5/07).

The association between health worker density and health outcomes has been examined in various studies, and it is generally accepted that, where health workers are scarce, health services and health outcomes suffer. For example, countries with low ratios of health workers to population are among the countries with high mortality rates for children under five years of age (Figure 2).

The challenges in maintaining an adequate health workforce that meets the needs of a population with social, demographic, epidemiological and political transitions require a sustained effort in addressing workforce planning, development and financing. Further examination and analysis are needed to better understand the factors that contribute to health worker retention in resource-constrained settings and the initiatives that have the potential to maintain a competent and motivated health workforce in Pacific and Asian countries (See Figures 1 and 2).

### To leave or to stay in the health workforce?

#### Decision-making factors

Skilled health workers are increasingly taking up job opportunities in the global labour market as the demand for their expertise rises in high-income areas. The rural to urban, intraregional and international migration of health workers in Asian and Pacific countries inevitably leaves poor, rural and remote areas under serviced and disadvantaged.

While some countries, such as India, Indonesia and the Philippines, have specifically trained health professionals for export to developed countries, the unplanned loss of health workers can be extremely costly due to their lengthy education programs, the high cost of teaching materials and techniques, and the need to hire replacements that may lack appropriate skills, languages or cultural sensitivity [4]. When migrants leave their positions in search of better opportunities, many have the intention of sending a portion of their income back to their families. For some countries, the value of these remittances is among the most stable sources of external finance, even exceeding the official development aid flow [5]. A study of Tongan and Samoan nurses in Australia found that their remittances to their home countries far outweighed the cost of training replacement nurses [6].

**Figure 3:**
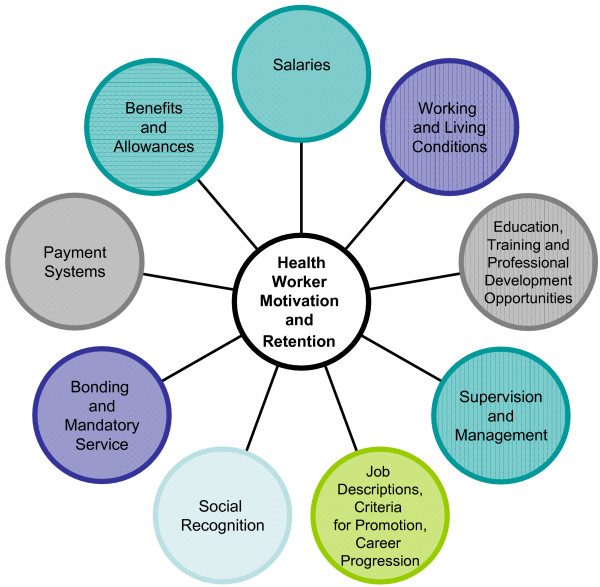
Factors affecting health worker motivation and retention.

While economic factors play a large role in health worker motivation and retention, they are not the sole reasons for health worker shortages (Figure 3). Health workers leave their positions for numerous reasons (Table 1). Surveys of health workers in five Pacific countries examined reasons for leaving or staying in their country of origin and demonstrated that there are common patterns among countries, even though there is variation in the relative importance of factors influencing individuals [4]. Findings indicate that health workers commonly leave to obtain better salaries, training opportunities and more desirable working conditions, to access education for children, to find political stability, and because of family ties abroad. Evidence from the same studies indicate that health workers who remain in their countries of origin hold more senior positions, receive good salaries and privileges, and work in favoured locations (See Figure 3 and Table 1).

**Table 1:** Reasons for job dissatisfaction and leaving the health workforce

Low salaries	Fiji, Samoa, Tonga, Vanuatu (WHO 2004) PNG (Bolger 2005) Vietnam (Dieleman 2005) Cambodia (Soeters 2003, Oum 2005) Thailand (Wibulpolprasert 2003)
Lack of adequate allowances	Fiji (WHO 2004) Vietnam (Dieleman 2005)
Poor working conditions	Fiji (WHO 2004) PNG (Bolger 2005) Vietnam (Dieleman 2005)
Inadequate facilities and shortages of drugs/equipment	Fiji, Samoa, Tonga, Vanuatu (WHO 2004) Cambodia (Oum 2005), Pakistan (Dussault 2006)
Difficult transportation	Vietnam (Dieleman 2005)
Weak support, supervision and management	Fiji, Tonga (WHO 2004) PNG (IMRG 2006) Vietnam (Dieleman 2005) Cambodia (Soeters 2003)
Heavy workload	Fiji, Samoa (WHO 2004) Vietnam (Dieleman 2005)
Mismatch in skills and tasks	Fiji, Vanuatu (WHO 2004)
Limited opportunities for professional development	Tonga (WHO 2004) Vietnam (Dieleman 2005)
Limited scope to upgrade qualifications	Fiji, Samoa, Tonga (WHO 2004) PNG (Bolger 2005) Vietnam (Dieleman 2005, Nguyen 2005) Pakistan (Adkoli 2006)
Lack of job prospects	India, Sri Lanka (Adkoli 2006)
Lack of promotion prospects/career structure	Fiji, Samoa (WHO 2004)
Inadequate living conditions	PNG (Bolger 2005)
Risk of violence/Lack of safety	PNG (Bolger 2005)
Political instability	Fiji (WHO 2004), Pakistan (Adkoli 2006)
Family members living abroad	Samoa (WHO 2004)
Education prospects for children	Fiji (WHO 2004)

The shortage of skilled health workers in many Pacific and Asian countries is compounded by the difficulties in training adequate numbers of health workers and balancing the skill mix and distribution in a country. Health workers have been reluctant to work in rural and remote areas because of little support or supervision, a lack of material resources for health, poor working and living conditions, and isolation from professional colleagues. Developing countries often experience 'urban bias' – where the political and economic forces support the provision of services and investment in urban areas to the detriment of rural areas. This increases the disparities in health worker distribution, access to services, and health outcomes [7].

A survey of 234 health providers in rural Vietnam – where approximately 75 per cent of the total population and 90 per cent of the poor live – demonstrated the low quality of both public and private health services in rural communities, and highlighted that 11 per cent of private providers had no qualifications [8]. Health workers with higher education levels in Vietnam tend to be in urban areas [9].

In the Pacific region, doctors are generally employed in hospitals in urban areas, while nurses deliver the majority of health services in rural areas. For example, more than 50 per cent of all doctors in Papua New Guinea work for the National Department of Health (including urban clinics in the National Capital District), approximately 37 per cent work in hospitals and less than 10 per cent work in the provincial areas, while over half of all nurses work for provincial health services [10].

In Cambodia, there is a poor distribution of doctors as well as an acute shortage of midwives outside the capital city, particularly in remote areas and sparsely populated communities [11].

To attract and retain health workers in rural and remote communities, innovative strategies are required.

#### Coping strategies

Health workers respond to inadequate or intermittent remuneration, poor working conditions and poor supervision with various coping strategies. For example, health workers may engage in 'dual practice', or hold multiple jobs in both the public and private sectors. Though dual practice is condoned in many countries, there is a risk that it can negatively influence the quality of care of the public services as it may encourage health workers to skimp on their public health efforts and to make referrals to their own private practices. In Cambodia, health workers with very low and irregularly paid salaries are forced to seek alternative sources of income for their survival. Although dual practice is not authorized by legislation, the authorities do not object if public health workers open private clinics, laboratories or pharmacies [12]. Many health workers in Vietnam maintain a private practice next to the public health facility where they are employed [13].

Another coping strategy is over-prescribing drugs and diagnostic tests. This has been shown to be a problem in rural China where low utilization of health services has led to over prescribing in order to increase income from the regular clients [14]. Other coping strategies include pilfering public goods (drugs and supplies) to sell or use in private clinics, informal user fees and absenteeism.

**Figure 4:**
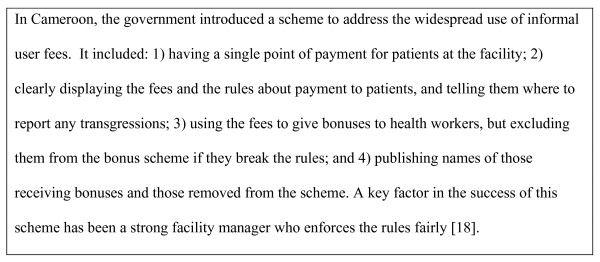
**Counteracting informal user fees**. Source: World Health Organization. The World Health Report 2006: Working Together for Health, 2006 [18].

To minimize the negative effects of coping strategies, the causes of health worker dissatisfaction must be addressed in workforce policy and planning (See Figure 4).

### Incentives for health worker retention and performance

#### Financial incentives: does money matter?

Financial incentives have been shown to be an important motivating factor for health workers, especially in countries where government salaries and wages are insufficient to meet the basic needs of health workers and their families. These incentives include higher salaries, salary supplements, benefits and allowances.

#### Higher salaries

Countries such as Fiji, Samoa, Tonga, Vanuatu, Papua New Guinea, Vietnam, Cambodia and Thailand have identified low salaries as a major reason for job dissatisfaction and/or migration among health workers [4,11-13,15,16]. Improved salaries and benefits are major financial incentives for workers to remain in the health sector. For example, since the mid-1990s Vietnam has encouraged doctors to work in communes in remote and disadvantaged areas by establishing permanent state staff positions with salaries and allowances from the state budget [9]. This measure has improved the overall numbers of medical doctors working at the commune level in Vietnam; however, there is wide variation between provinces. Findings from a survey in Bangladesh of one hundred government-employed doctors with private practices indicate that doctors in primary health care would give up private practice if paid a higher salary, while doctors in secondary and tertiary care reported a low propensity to give up private practice [17].

In resource-constrained settings, it is often difficult to increase salaries. In addition, the structure of public service salaries in some countries is not easily altered because of public expenditure ceilings or public service commissions that consider it unfair or unwise to raise salaries in one sector alone [18]. In East Timor the Ministry of Health wants to explore the use of incentives to compensate staff for working in remote and isolated conditions. However, this will require a whole-of-government approach, as staff ceilings and salaries are subject to strict civil servant regulations [19].

**Figure 5:**
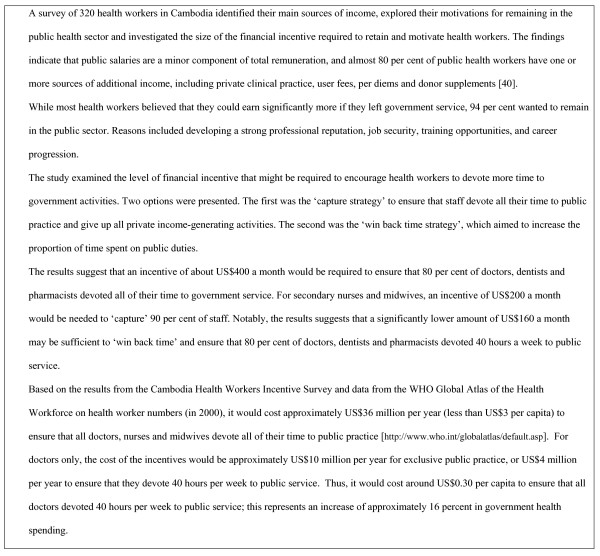
**Keeping Cambodian health workers in the public system: how much is needed?**. Source: Ministry of Health, Cambodia. Cambodia Health Workers Incentive Survey. 2005 [40], and WHO Global Atlas of the Health Workforce .

Countries unable financially to revise the pay scales for all health workers, yet have the flexibility to alter some salaries, may consider increasing the pay and benefits of high-priority groups. In Fiji, the government responded to a national nursing strike by revising the pay scale, reviewing minimum qualifications, developing fairer rostering, and implementing hardship allowances for nurses in rural areas [4]. In Thailand, the 1990s payment reforms for health workers in rural areas included supplements to doctors in eight priority specialties, combined with compensation for doctors, dentists and pharmacists not in private practice, and additional financial and non-financial incentives [18]. However, increasing the salaries and benefits of priority groups is a complex endeavour that must be determined carefully by government, since incentives aimed at one group of professionals may affect the entire system (See Figure 5).

It is virtually impossible for developing countries to compete with the salaries of developed nations. For example, specialist doctors in Sri Lanka were paid 45 000 rupees a year while their counterparts in Australia were paid the equivalent of 1.5 million rupees a year [20]. Salaries of public health personnel in Vietnam were very low, averaging US$ 29 a month [13]. Similarly, in Cambodia health workers received irregularly paid salaries of US$ 10–30 a month [12]. Therefore, when starting from such a low base, even significant improvements in salaries are likely to be only one part of the package of incentives that health workers consider when deciding whether to stay in the domestic workforce.

All remuneration strategies must be monitored and adapted over time to ensure that the desired outcomes are achieved.

#### Salary supplements, benefits and allowances

Countries have adopted various initiatives to mitigate the low remuneration in the public sector. These include financial allowances to attract and retain health workers such as the rural location/hardship allowance, the public sector retention allowance and the accommodation allowance. Additional financial benefits include overtime pay, pension plans, health/life insurance, contract gratuities, and transportation allowance. In Papua New Guinea, there is a Domestic Market Allowance, which is intended to assist in recruiting and retaining doctors and nurses when public service salaries are substantially lower than those prevailing in the domestic labour market [21,22].

In Thailand, special hardship allowances are provided as incentives for doctors to remain in rural areas. The allowance has three tiers based on location: rural districts, remote districts, and the most remote districts [16]. Doctors in the most remote districts received US$500 a month – almost three times their basic salary. A non-private practice allowance of US$ 400 a month was given to doctors who agreed not to engage in private practice, and special workload-related payments were implemented for service in non-official hours. In total, a new medical graduate working in a rural district received between US$ 825 a month (in regular districts) to US$ 1379 a month (in the most remote districts). But this was still lower than the salary of a new graduate working in private practice in an urban area, which was at least US$ 1500 a month.

The efficacy of using financial incentives to motivate and retain health workers in Pacific and Asian countries needs to be evaluated. Country-specific studies that examine health worker preferences, financial priorities and responses to financial incentives would assist governments to modify and refine benefits and allowances.

#### Donor assistance for salaries and innovative financial incentives

Harnessing international donor aid for salaries and innovative financial incentives is one way to overcome resource constraints. Traditionally, donors have been hesitant to contribute to national salaries or incentive packages because of concerns about sustainability and being able to track results linked to the financial inputs. The exceptions have been vertical programs such as national disease control programs where financial incentives have been common practice and are considered to be a key to the success of these interventions [23].

One question that deserves discussion is whether development partners should reconsider their reluctance to provide funding for salary incentives. If health worker performance is limiting the effectiveness of development partners' inputs to health, it may be a sensible investment to provide incentives for performance. The issue of sustainability may be irrelevant in centres that will be dependent on external assistance for many years ahead.

**Figure 6:**
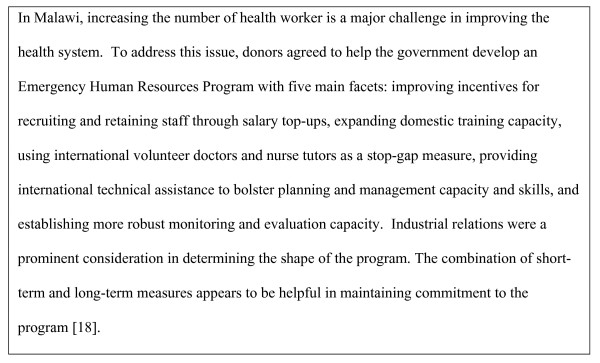
**Donor assistance for salaries and incentives in Malawi**. Source: World Health Organization. The World Health Report 2006: Working Together for Health, 2006 [18].

In recent times, there has been a shift among some development partners towards funding to cover wages [24]. For example, in Malawi, donors collectively recognized that the lack of human resources was a serious constraint on the success of donor-funded projects and decided to support financial incentives for health workers. This action was considered an 'exceptional measure that might otherwise be deemed unsustainable' [25] (See Figure 6).

In Cambodia, the government and development partners implemented the Merit Based Payment Initiative in 2005 within the Ministry of Economy and Finance, with plans to expand to other ministries including the Ministry of Health. The program rewards civil servants with higher pay in accordance with their merit, and is accompanied by a rigorous performance management system. At present, the government is bearing 11% of costs, with its share increasing each year to reach 35% by 2011 [26]. In addition to these innovative schemes, financing mechanisms such as the Global Fund to Fight AIDS, Tuberculosis and Malaria have allowed often generous salary supplements to be paid to government health workers.

#### Non-financial incentives: what else is needed?

Several studies have shown that financial incentives alone are not sufficient for retaining workers in the health sector [4,5,27]. According to an analysis by Vujicic et al. on the role of wages in the migration of health professionals from developing countries, the wage differentials between source and destination countries are so large that small increases in wages in the developing countries are unlikely to make a significant difference to migration patterns [27]. A qualitative study of doctors in Samoa revealed that several doctors received regular pay increases, pensions and housing allowances, and appeared to be relatively satisfied with their jobs. However, due to their long working hours, overburdened workloads, inadequate pay structures and a large number of family members living overseas, migration remained an attractive option [4].

A range of non-financial incentives are needed to complete a package that will attract health workers – especially to rural and remote areas – and encourage them to stay in the workforce. They include the broad categories of improved working and living conditions, continuing education, training and professional development, improved supervision and management, and gender-sensitive considerations.

#### Improved working and living conditions

The working environment has a strong influence on job satisfaction. Decisions by nurses and doctors to migrate are often related to a poor working environment [4,13,15]. All workers require adequate facilities and conditions to do their jobs properly. While most evidence is anecdotal, the benefits of improving working and living conditions appear to be significant. It is generally understood that health workers value working conditions that include appropriate infrastructure, water, sanitation, lighting, drugs, equipment, supplies, communications and transportation. A study in Bangladesh revealed that remoteness and difficult access to health centres were major reasons for health worker absenteeism, while health personnel working in villages or towns with roads and electricity were far less likely to be absent [18].

Safe working and living conditions also contribute to worker satisfaction. Safety is an important factor in countries such as Papua New Guinea, where the risk of violence is high [15]. Violence against female health workers, including physical assaults and bullying, is a particular problem worldwide. In Tonga, security was an issue for nurses posted to remote locations [4]. Some research findings suggest a direct link between aggression in the workplace and increased sick leave, burnout and staff turnover [18]. Holistic strategies to prevent workplace violence can be complex and costly. However, some measures that may be implemented in resource-constrained settings include policies that require health workers to operate in teams, community watch and alert mechanisms, improvements in the layout of health centres, and the use of private rooms. A clearer understanding of health worker needs can contribute to initiatives to improve working and living conditions in a particular area.

#### Continuing education, training and professional development

Opportunities to continue education, training and professional development have been identified as important motivating factors for health workers. Programs that focus on local conditions, including training in local languages and in skills that are relevant to local needs, can help to limit workforce attrition [18]. In addition, maintaining appropriate regional standards may assist with the distribution of health workers. The Pacific Islands Forum Secretariat and the World Health Organization are considering the possibility of enhancing and standardizing regional training programs across the Pacific [28].

The provision of specialized training is difficult in countries where resources are limited and training opportunities are scarce. A way of improving training opportunities, which was suggested by the WHO migration study, involves using open learning courses to provide updated knowledge to medical staff [4]. Findings from Fiji suggest that this would alleviate the need for doctors to travel overseas to study, making it less likely to 'lose' them as a result of a combination of favourable overseas experiences and a lack of job satisfaction at home.

The lack of professional development has been cited as a reason for job dissatisfaction [4,13,15]. This is especially true of health workers in rural or remote areas who are often isolated from professional colleagues and support. A qualitative study of rural midwives in Australia illustrates that continuing professional development and an organizational culture of ongoing learning are considered to be important strategies for the retention and professionalism of midwives [29]. In the Pacific region, most continuing professional development is funded by the fees health workers pay to professional associations. However, membership numbers of these associations are often insufficient to enable viable programs on a regular basis [28]. Some incentives to improve professional development are included in health worker benefits. For example, in Papua New Guinea, senior medical officers are entitled to receive a six-month sabbatical for training and refresher courses every four years [21]. Research is needed to ascertain the extent to which such incentives influence the motivation and retention of health workers.

#### Rural recruitment and placement

Improving the distribution of health workers within a country requires attracting health workers to rural and marginal communities and retaining them there [1]. Studies in the United States and Canada have shown that health workers with a rural background, a preference for life in smaller communities, and education in rural medicine are likely to be both recruited for and retained in rural communities [30-32].

In East Timor, recruiting midwives for remote areas is difficult. As a result, the Ministry of Health has started a midwifery course where female nurses currently working in (or with strong links to) rural areas with vacancies are selected and trained for an additional year in midwifery and then posted to these priority areas [19]. To improve the distribution of nurses, midwives and doctors, Thailand has used rural recruitment, training in rural health facilities, hometown placement and contractual agreements [16]. Students receive highly subsidized education as well as free clothing, accommodation, food and learning materials as incentives. To retain health workers in rural areas for the long term, the study has shown that recruitment should be restricted to those who were raised in the rural areas, thus excluding individuals who relocated to rural areas two or three years before enrolment in the hope of being recruited.

#### Rotation from rural and remote posts

Research findings suggest that health workers in rural areas should received scheduled rotations to prevent extended professional isolation. In Vanuatu and Samoa, as in other countries with shortages of health workers, those in rural and remote areas face a lack of supervision, poor working conditions, a lack of supplies, poor transportation and communication, and a lack of support, all of which increase job dissatisfaction and the potential for urban or overseas migration. The fear of an indefinite posting to these areas can hinder recruitment.

Qualitative research on overseas-trained doctors in rural New Zealand revealed a theme of physical and social 'entrapment' arising from their isolation [33]. This isolation diminished their liking for rural placement and led practitioners to consider leaving. A study from Tonga showed that nurses were rotated more regularly between hospitals, departments, and rural and urban clinics than their counterparts in other Pacific countries [4]. This was found to be particularly important in preventing burnout, as well as in increasing their development and sharing of skills.

#### Improved supervision and management

**Figure 7:**
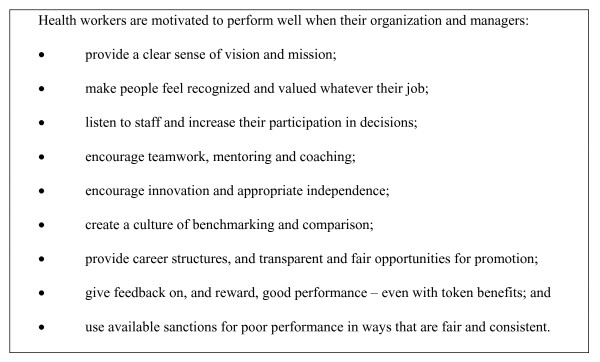
**The importance of good supervision and management**. Source: World Health Organization. The World Health Report 2006: Working Together for Health, 2006 [18].

Good supervision and management – including adequate technical support and feedback, recognition of achievements, good communication, clear roles and responsibilities, norms and codes of conduct – are critical to the performance of health systems and the quality of care [18]. Weak support, supervision and management have been identified as factors in job dissatisfaction in many countries, including Fiji, Tonga, Papua New Guinea, Vietnam and Cambodia [4,12,13,34] (See Figure 7).

Management strategies to increase recognition and social acceptance of health workers have been shown to increase job satisfaction and motivation. A study of rural health workers in northern Vietnam revealed that appreciation by managers, colleagues and the community was a major motivator. However, positive feedback was lacking when the health workers performed well, and staff appraisals were considered to be for administrative purposes rather than performance improvement [13]. The study showed that management tools to motivate health workers were not optimally implemented. In Thailand, the establishment of a rural professional society – the Rural Doctor Society – improved the skills of health managers and enhanced the social recognition of health workers and, hence, their job satisfaction [16]. Another strategy to improve the responsiveness and effectiveness of health workers involves increasing community participation [35]. Measures such as exit surveys on the quality of care received in a health facility may increase their engagement and support from local communities.

**Figure 8:**
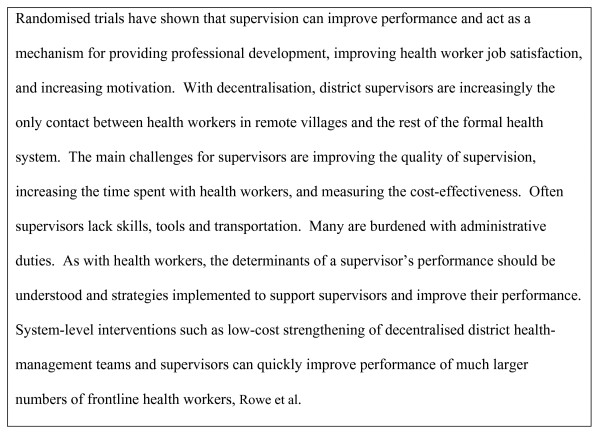
**Can supervision improve health worker performance?**. Source: Rowe AK, de Savigny D, Lanata CF, Victora CG: How can we achieve and maintain high-quality performance of health workers in low-resource settings? Lancet, 2005; 366:1026–35.

Human resource management tools comprise the policies, practices and activities at the disposal of managers to obtain, develop, use, evaluate, maintain and retain the appropriate number, skills mix and motivation of employees to accomplish the organisation's objectives [36]. These tools form the basis for improving management, together with monitoring and evaluation systems that link health worker performance to supportive supervision and appraisal. Ultimately, these systems should be linked to criteria for promotion and career development. An effective management system needs to have the capacity to regularly assess the performance of health workers and the engagement a well-trained manager. While this may be difficult in rural and remote areas where supervision and management are weak, simplified systems can be developed, drawing on health workers themselves to assist in designing a system. (See Figure 8)

#### Job descriptions, criteria for promotion and career progression

There is a positive association between the performance of health workers and the clarity of their job descriptions. A survey of Indonesian nurses and midwives found that approximately 47% of did not have job descriptions and 40% were engaged in work other than nursing or midwifery [18]. Based on survey results, clear job descriptions and a performance monitoring system were developed and implemented. Staff reported that the job descriptions together with standards of operation and procedures had given them greater confidence about their roles and responsibilities. It is important that health workers have their skills matched to their tasks. In Vanuatu, well-qualified nationals with postgraduate qualifications have returned to the country to take up specific positions, only to be redeployed to duties that are not directly related to their expertise and training [4].

Transparent mechanisms for promotions and rewards are also important. In Vietnam, a study of rural health workers demonstrated that those seeking to upgrade their skills through training for a diploma or certificate did not understand the criteria for the selection of candidates and therefore felt that the process was arbitrary [13]. In Nepal, health workers in rural areas were critical of a policy that offered the potential for sponsored higher education abroad but did not link these opportunities to performance [37]. Better information, communication, job descriptions, accountability and criteria for rewards could increase transparency and health worker motivation.

#### Potential for dual practice

One way of attracting and retaining skilled health workers in the public sector is to permit dual practice when public salaries and wages are substantially lower than in the private sector. Although there are concerns about insufficient time and effort devoted to public practice along with the potential for referrals to the private sector and pilfering of public goods, the arguments for allowing dual practice include [38]:

• the supply of health providers willing to work in the public sector is higher than it would be if the providers were not allowed to augment their low public salaries with private earnings,

• providers have an incentive to perform better in order to gain a good reputation and attract patients to their private practices, and

• providers may enhance their technical knowledge and skills through exposure to multiple practice settings.

In Vietnam, Cambodia and Indonesia, it is widely accepted that public health workers maintain a private practice to subsidies their government incomes [12,13,39]. In Indonesia, more than 80% of public doctors are involved in some form of private practice [38]. In Phnom Penh, Cambodia, 90% of a doctor's total income from dual practice is derived from the private sector, while in Thailand, doctors' earnings from private practice constitute 55% of their total income [39].

Studies have shown that there are both financial and non-financial benefits for health workers that work in both public and private sectors. A survey of 100 public doctors in Bangladesh suggested that dual practice allows health workers to retain the status of a government job while minimizing opportunity costs and economic losses [17]. Similarly, a study in Cambodia found that dual practice is an attractive arrangement that ensures that health workers can maintain a strong professional reputation, job security, training opportunities and career progression from their public positions while increasing their earnings from the private sector [40]. More than half of the dual practitioners in the study felt that their public positions increased their earnings from private practice due to increased prestige.

Though dual practice is an incentive for many health workers worldwide, few studies have analysed the complex relationships and conflicting interests that emerge. An analysis of dual practice in the health sector by Ferrinho and Van Lerberghe (2004) states that there is 'no evidence that dual practice by public sector health professionals complements public practice or promotes greater equity of health care distribution' [39]. The potentially negative consequences of permitting dual practice as a way to retain health workers should be considered carefully prior to its inclusion in any incentives package. In addition, formal instruments for monitoring and sanctioning penalties are needed to enforce rules and regulations such as after-hours private practice in public health institutions [18].

#### Gender considerations

**Figure 9:**
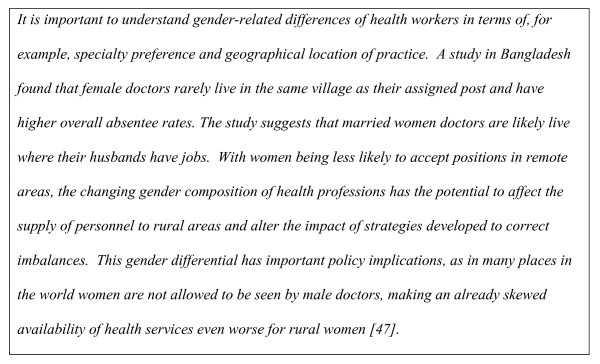
**Female practitioners in the health workforce**. Extracted from: Dussault et al. Not enough there, too many here: Understanding geographical imbalances in the distribution of the health workforce. Human Resources for Health, 2006 [47].

In the majority of countries, women are the primary caregivers. As women make up an increasingly large proportion of the health profession, it is important to consider the different needs of female health workers when developing incentives. Flexible and/or part-time working hours, flexible leave/vacation time, access to child care and schools, and planned career breaks are a few of the incentives that may be important to female health workers. A survey of 271 female general practitioners and 31 specialists in rural Australia found that 36% of general practitioners and 56% of specialists would prefer to work fewer hours [41]. Results indicated that incentives to attract and retain women in rural practice include flexible practice structures, acceptance of the rural area by the doctor's family, mentoring by women doctors, and financial and personal recognition (See Figure 9).

### Approaches to incentives for health workers

#### Performance-based incentives

According to Bandaranayake, performance management aims to optimize the quality of work and efficiency of the health system through quality assurance strategies and surveillance mechanisms. It reflects the overall vision, aims and objectives of the organization, the lines of accountability, and a clear understanding of how the individual or team can best contribute. It also includes career planning or personal development and may be linked to an incentive scheme [42].

**Figure 10:**
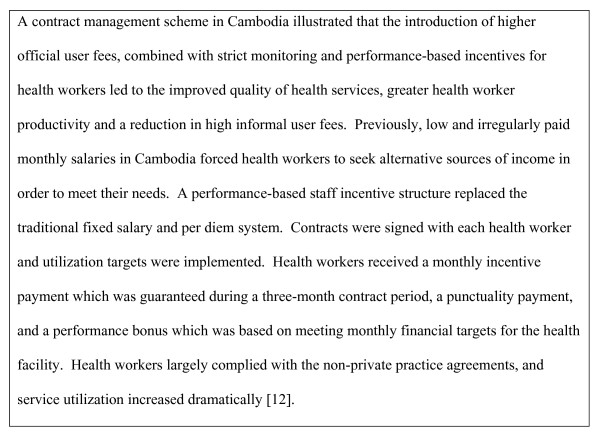
**Performance-based incentives for health workers in Cambodia**. Source: Soeters R, Griffiths F. Improving government health services through contract management: a case from Cambodia. Health Policy and Planning, 2003 [12].

Performance-based incentives are receiving increasing interest from health systems worldwide, though evidence on the effectiveness of these incentives in Pacific and Asian countries is limited. In Sri Lanka, performance-based non-financial incentives such as career development, training opportunities and fellowships were found to be appropriate for central and provincial managers, while hospital managers preferred financial incentives [42]. In Cambodia, performance-based financial incentives for health workers led to better quality health services, increased health worker productivity and reduced informal user fees (See Figure 10).

It is important to recognize that the use of incentives to improve performance typically requires good regulatory frameworks and skilled managers [43]. These are often deficient in developing countries. Measuring performance outcomes against quantified objectives is difficult where management capacity is weak and health information systems are not well developed. Where the health sector is severely under-resourced it is difficult to hold people accountable for how they do their jobs [7]. In a study of twelve developing countries (including Cambodia, Indonesia, Myanmar, Papua New Guinea and Vietnam) that adopted innovative strategies for improving health services and systems, it was found that the introduction of performance incentives for health workers was unlikely to be successful because of the lack of resources to finance and monitor the implementation [44].

Health workers must be well informed about the performance objectives, the criteria for meeting those objectives, the use of monitoring tools/systems, and the resulting incentives or disincentives that are based on their performance. For performance-based incentives to be successful, there must be standard measures or baselines against which performance is monitored, comparisons are made, and improvements are recommended. For individuals, measures may include punctuality, productivity, attitude and achievement of objectives on time. In some settings in the Asia-Pacific region, it may be more practical and culturally acceptable to offer incentives to teams rather than individuals. Performance management for teams must be built on group identities, with awards designed for teams [36]. Measures of performance may include group productivity, motivation and achievement of objectives. Health system measures may include service utilization and quality-of-care indicators.

Many performance measures are outcome oriented, and therefore do not provide an indication of the process by which the outcomes are achieved. A study in China of the effect of performance-related pay for hospital doctors on hospital behaviour found that the 'bonus system' led to improved productivity and cost recovery. However, there was an increase in unnecessary care and admittance of patients, as well as an over prescription of drugs [45]. It is important to design performance monitoring systems in a way that does not result in undesired outcomes. Ideally, a performance-based incentive system should include monitoring of both process and outcomes.

Before implementing performance-based incentives, it is essential that managerial staff is committed to the system. Such incentives are of little use without managers willing and empowered to act on results [42]. To apply the incentives and monitor performance requires trained technical staff with analytical skills and strong managerial qualities. It is critical to understand the local management culture, particularly in Pacific and Asian countries where cultural and kinship practices influence many aspects of the national government, and where the system of automatic promotion based on seniority is deeply embedded.

#### Strategies for return migration

**Figure 11:**
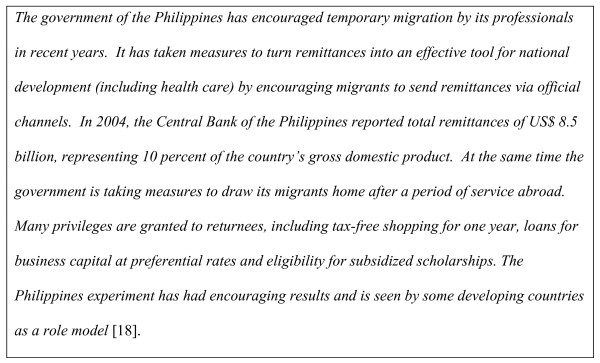
**Turning brain drain into brain gain – the Philippines**. Excerpt: The World Health Report 2006: Working Together for Health. World Health Organization, 2006 [18].

Various strategies to encourage return migration have been tried in Pacific and Asian countries. A study in Tonga has shown that many skilled returnees apply their skills on return to the country [4]. Strategies to facilitate the return of migrants have been implemented in the Cook Islands using the establishment fund and family incentive scheme [46]. The Philippines has been successful in getting skilled migrants to return and put their skills to use (See Figure 11).

Continued research and evaluation of incentives for migrants to return are needed in Pacific and Asian countries to understand the extent of return migration, its components and rationale.

#### Restrictive measures and sanctions

Restrictive measures – such as mandatory service – can be effective means of retaining health workers, though they require monitoring and management to ensure adherence and to apply penalties when necessary.

In Indonesia, after the completion of compulsory public service, health workers who work in very remote areas receive a higher salary and the guarantee of a civil service career that is highly desirable since it allows for private practice in the evenings as well as free access to specialist training [47]. Unfortunately, it was noted that individuals who are interested in specialist training often have no interest in public health, and thus leave the rural areas soon after the completion of the compulsory contract.

In Thailand, doctors are required to fulfil three years of compulsory public service after finishing their training and must pay a fine if they breach the contract [16]. Another restrictive measure used in Thailand is a prerequisite of at least one year of public service in a rural area before specialist training can be undertaken [16].

Bonding and mandatory service requirements for recipients of government scholarships have been tried over the years by Pacific countries with limited success [46]. Ministers of Health from Pacific island countries, together with WHO and the Secretariat of the Pacific Community, recently developed 'The Pacific Code of Practice for Recruitment of Health Workers in the Pacific Region' to provide a framework for better managing the loss of skilled health workers through migration [48]. An important element of the code is ethical recruitment that includes fulfilling contractual obligations, such as a bond to the government for those who benefited from national scholarships, prior to international recruitment.

Sanctions can be difficult to enforce where management and monitoring capacity are poor, and where cultural, kinship or hierarchical systems prevent the unbiased application of rules and regulations to all health workers. An example is the 'wontok' system of loyalty found in Solomon Islands, Papua New Guinea and other Melanesian countries. The system is built on the premise that loyalties to kin supersede all other loyalties. This adds a layer of complexity to policy coordination as decision making at the national level must be balanced with the role of village elders or chiefs in the Pacific [49]. The wontok system may prevent managers of health workers from regulating the behaviour of their staff. In Cambodia, the contracting experiment to improve the performance of health workers proved to be successful, yet the results indicate that there were problems in sanctioning penalties even though few violations were documented [12]. Effective incentive systems require regulation and governance structures that minimize problems of patronage and corruption [43].

#### Packaging financial and non-financial incentives

A workforce's motivation and performance typically result from a package of linked incentives, rather than from individual measures. For such packages to be effective, the incentives must be based on the local context and the organisation's structure, culture and institutional capacity, the wider social values and expectations, the ease of implementation and monitoring, the cost and timeframe for the package to take effect, and the sustainability of the package [18].

Evidence indicates that for health workers both financial and non-financial incentives should be considered. A qualitative study of what motivated rural health workers in Vietnam identified appreciation, job stability, regular income and continuing education as the main motivating factors, and low income and allowances as the main discouraging factors [13]. The response of health workers to incentives also depends on their career stage, experience level, and social/familial responsibilities [43]. A study of doctors in Bangladesh found that financial incentives that aim to increase the number of doctors in rural areas, such as a non-private practice allowance, are more likely to be appreciated by doctors who are at the beginning of their career [17]. Ideally, incentives structures should recognize the different stages in health workers' careers and the various expectations at each stage.

The introduction of any package of incentives designed to attract and retain health workers must be accompanied by continuous monitoring and assessment of its effectiveness – together with research on factors that motivate health workers – in order to adapt and adjust the package to the changing needs and desires of the workforce. For many Pacific and Asian countries this means that the incentive packages must be simple enough to be easily managed and monitored, and may exclude complex systems for monitoring performance.

**Figure 12:**
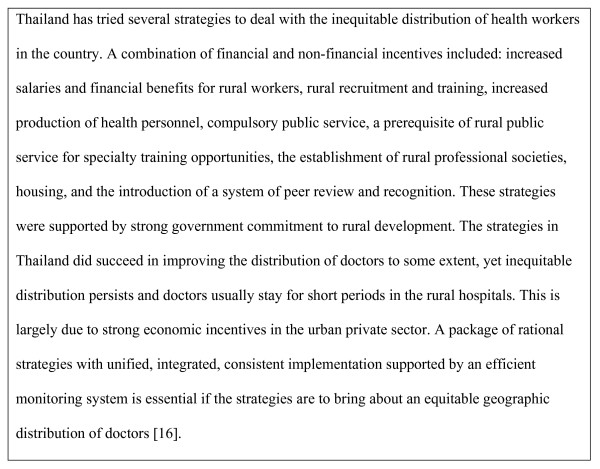
**Integrated strategies to tackle the inequitable distribution of doctors**. Source: Wibulpolprasert S, Pengpaibon P. Integrated strategies to tackle the inequitable distribution of doctors in Thailand: four decades of experience. Human Resources for Health, 2003 [16].

In theory, it is easier to design incentive packages for health workers in a decentralized system [43]. However, this is not necessarily the case in developing countries (such as Papua New Guinea) because of the lack of capacity at subnational levels. In Samoa and Fiji, the World Bank suggested that a human resource plan was needed to provide incentives to improve staff performance, including attractive salaries, in-service training programs linked to salary increments, well-structured career development paths and performance-based rewards [4]. While these are aspects of a comprehensive human resource plan that countries should ultimately strive to achieve, many developing countries do not have the resources to implement such plans. Early attempts to implement incentive packages in countries with limited capacities may be more successful by selecting incentives that can be easily monitored without complex administrative systems (See Figure 12).

## Conclusion

The shortage of health workers in Pacific and Asian countries is a critical issue that must be addressed as an integral part of strengthening health systems. Health workers migrate, leave the health sector, or use various coping strategies in response to difficult circumstances such as poor or intermittent remuneration, inadequate working conditions, limited training opportunities or weak supervision.

To minimize attrition from the health workforce and the negative effects of coping strategies, efforts are required to address the causes of health worker dissatisfaction and to identify the factors that influence health worker choices. The challenges in maintaining an adequate health workforce require a sustained effort in workforce planning, development and financing. This effort requires innovative strategies – such as incentive packages – for retaining and motivating health workers in resource-constrained settings.

The health system in each country is different and requires different strategies to stem the loss of skilled health workers, especially in rural and remote areas. Consequently, there is no global model for improving the retention of health workers and their performance. The literature highlights the importance of considering a broad range of incentives that may be packaged to attract health workers and to encourage them to stay in the health sector. It emphasizes that non-financial incentives can be as crucial as financial incentives.

There is potential for health worker incentives schemes to succeed in the Asia-Pacific region. Successful incentive strategies are multifaceted and include:

• long-term political commitment and sustained effort at all levels

• a deep understanding of the cultural, social, political and economic context in which the incentives strategy is being developed

• involvement of key stakeholders – especially the health workers themselves – in developing the strategy, formulating policy and implementing initiatives

• integration of efforts between government sectors, donors, non-governmental organizations and the private sector to ensure the initiatives are sustainable

• packages of coordinated and linked financial and non-financial incentives that adequately respond to the needs of health workers

• monitoring and evaluation tools and systems

• strengthened supervision and management capacities

• performance management systems that link health worker performance to supportive supervision and appraisal, and

• continued research on what motivates health workers in order to adapt and adjust the incentives to the changing needs and desires of the workforce.

While the literature identifies several approaches for improving the motivation and retention of health workers through the use of incentives, there is a paucity of evidence on the efficacy of various incentives schemes. Further examination and analysis are needed to better understand the contributing factors to health worker motivation and retention, and to ascertain the extent to which different incentives, or packages of incentives, influence health workers. This information is critical for effective workforce planning and policy development in the health sector.

In conclusion, incentive packages to attract, retain and motivate health workers should be embedded in comprehensive workforce planning and development strategies in Pacific and Asian countries. Research findings from the region indicate that improved salaries and benefits, together with improved working conditions, supervision and management, and education and training opportunities are important. Country-specific strategies require examination of the underlying factors for health worker shortages, analysis of the determinants of health worker motivation and retention, and testing of innovative initiatives for maintaining a competent and motivated health workforce. Continued research and evaluation will strengthen the knowledge base and assist the development of effective incentive packages for health workers. For additional reading please see Additional file 1.

## List of abbreviations used

AAAH: Asia Pacific Action Alliance on Human Resources for Health; ADB: Asian Development Bank; AHPSR: Alliance for Health Policy and Systems Research; AusAID: Australian Agency for International Development; DfID: United Kingdom Department for International Development; GHWA: Global Health Workforce Alliance; ILO: International Labour Organization; NZAID: New Zealand Agency for International Development; UN: United Nations; UNDP: United Nations Development Program; UNICEF: United Nations Children's Fund; USAID: United States Agency for International Development; WHO: World Health Organization; WB: World Bank.

## Competing interests

The authors declare that they have no competing interests.

## Authors' contributions

LH and JT collaborated on the design and drafting of the manuscript. All authors approve the final manuscript.

## Supplementary Material

Additional File 1 Additional reading Click here for file
