# Supplementary material for: Incentives for retaining and motivating health workers in Pacific and Asian countries
Source: Hum Resour Health. 2008 Sep 15;6:18. doi: 10.1186/1478-4491-6-18 (PMC2569066; doi:10.1186/1478-4491-6-18)
Supplement: Additional File 1 — Additional reading [file 1478-4491-6-18-S1.doc]

# Additional file 1 – Additional Reading

Agyepong IA, Anafi P, Asiamah E, Ansah EK, Ashon DA, Narh-Dometey C: Health worker satisfaction and motivation in the public sector in Ghana. International Journal of Health Planning and Management 2004, 19:329-336.

Alliance for Health Policy and Systems Research: Human Resources for Health: Evidence from systematic reviews of effects to inform policy-making about optimizing the supply, improving the distribution, increasing the efficiency and enhancing the performance of health workers. December, 2006.

Baumann A, Yan J, Degelder J, Malikov K: Retention Strategies for Nursing: A Profile of Four Countries. Human Health Resources Series, Nursing Health Services Research Unit, November 2006.

Benjamin TB: Factors influencing family physicians to enter rural practice. Does rural or urban background make a difference? Can Fam Physician 2005, 51(9): 1247.

Bennett S, Franco LM: Public Sector Health Worker Motivation and Health Sector Reform: a Conceptual Framework. Major Applied Research Technical Paper. Partners for Health Reform, 1999.

Dambisya YM: A review of non-financial incentives for health worker retention in East and Southern Africa. Regional Network for Equity in Health in East and Southern Africa (EQUINET) Discussion paper, No. 44, 2007.

Dieleman M, Toonen J, Toure H and Martineau T: The match between motivation and performance management of health sector workers in Mali. Human Resources for Health 2006, 4:2.

Disease control priorities project: Improving quality of clinical care: incentives for health workers. DCPP, 2006.

El-Jardali F, Jamal D, Abdallah A, Kassem K: Human resources for health planning and management in the Eastern Mediterranean region: facts, gaps, and forward thinking for research and policy. Human Resources for Health 2007, 5:9.

Furth R: Zambia pilot study of performance-based incentives. Quality Assurance Project, USAID, 2006.

García-Prado A, Chawla M: The impact of hospital management reforms on absenteeism in Costa Rica. Health Policy Planning, 2006.

Gonzélez P: Should Physicians' Dual Practice Be Limited? An Incentive Approach. Universidad de Alicante and CORE, 2002.

Hammer J, Jack W: Developing Incentives for Rural Health Care Providers in Developing Countries. World Bank, 2001.

Harries AD, Salaniponi F: Improving health workers' performance in low-resource settings. Lancet 2005, 366:1606.

Hongoro C, McPake B: How to bridge the gap in human resources for health. Lancet 2004, 364:1451-56.

International Council of Nurses: Nurse wages and their context. Database summary. ICN, 2005.

Jones JA, Humphreys JS, Adena MA: Rural GPs' ratings of initiatives designed to improve rural medical workforce recruitment and retention. Rural and Remote Health 4, 2004: 314.

Kearns R, Myers J, Adair V, Coster H, Coster G: What makes 'place' attractive to overseas-trained doctors in rural New Zealand? Health Soc Care Community 2006, 14(6), 532-540.

Kenyon M, Chevalier C, Gagahe V, Sisiolo R: The community in the classroom: Designing a distance education community health course for nurses in Solomon Islands. Pacific Health Dialog, 2000.

Kolehmainen-Aitken RL: Decentralization and its impact on the health workforce: perspectives from managers, workers and national leaders. Human Resources for Health 2004, 2:5.

Kotzee T, Couper ID: What interventions do South African qualified doctors think will retain them in rural hospitals of the Limpopo province of South Africa? Rural and Remote Health 2006, 6:581.

Lipinge S, Hofnie K, Van der Westhuizen L, Pendukeni M: Perceptions of health workers about conditions of service: a Namibian case study. EQUINET: Network for Equity in Health in Southern Africa, Discussion paper 35, May 2006.

Manongi RN, Marchant TC, Bygbjerg IC: Improving motivation among primary health workers in Tanzania: a health worker perspective. Human Resources for Health 2006, 4:6.

Martineau T, Lehmann U, Matwa P, Kathyola J, Storey K: Factors affecting retention of different groups of rural health workers in Malawi and Eastern Cape Province, South Africa. Liverpool School of Tropical Medicine, Final Report, 2006.

Noree T, Chokchaichan H, Mongkolporn V: Abundant for the few, shortage for the majority: The inequitable distribution of doctors in Thailand. Asia Pacific Action Alliance on Human Resources for Health country reviews, 2005. [http://www.aaahrh.org/docs.php]

Speybroeck N, Kinfu Y, Dal Poz MR, Evans DB: Reassessing the relationship between human resources for health, intervention coverage and health outcomes. In WHO Background papers for The World Health Report 2006. [http://www.who.int/hrh/documents/en/]

Ssengooba F, Rahman SA, Hongoro C, Rutebemberwa E, Mustafa A, Kielmann T, Mcpake B: Health Sector Reforms and Human Resources for Health in Uganda and Bangladesh: Mechanisms of Effect. Human Resources for Health 2007, 5:3.

Troy PH, Wyness LA, McAuliffe E: Nurses’ experiences of recruitment and migration from developing countries: a phenomenological approach. Human Resources for Health 2007, 5:15.

Van Lerberghe W, Conceicao C, Van Damme W, Ferrinho P: When staff is underpaid: Dealing with the individual coping strategies of health personnel. In Bulletin of The World Health Organization 2002, 80:581-584.

Vujicic M, Zurn P: The dynamics of the health labour market. International Journal of Health Planning and Management 2006, 21:101-115.

Watters D, Scott D: Doctors in the Pacific. MJA Volume 181 Number 11/12. December 2004.

World Health Organization: Improving Health Workforce Performance: In Search of Promising Practices. WHO, 2006.

World Health Organization and World Bank: Improving Health Workforce Performance. WHO, WB 2003.

World Health Organization Western Pacific Region: Regional Strategy on Human Resources for Health, 2006-2015. WHO Western Pacific Region, 2006.

Zurn P, Dal Poz MR, Stilwell B, Adams O: Imbalance in the health workforce. Human Resources for Health 2004, 2:13.
